# Real-Time Nitrate Ion Monitoring with Poly(3,4-ethylenedioxythiophene) (PEDOT) Materials

**DOI:** 10.3390/s23177627

**Published:** 2023-09-03

**Authors:** Michael C. Kohler, Fang Li, Ziqian Dong, Reza K. Amineh

**Affiliations:** 1 Department of Electrical and Computer Engineering, New York Institute of Technology, College of Engineering and Computing Sciences, Old Westbury, NY 11568, USA; mkohler@nyit.edu; 2 Department of Mechanical Engineering, New York Institute of Technology, College of Engineering and Computing Sciences, Old Westbury, NY 11568, USA; 3 Department of Electrical and Computer Engineering, New York Institute of Technology, College of Engineering and Computing Sciences, New York, NY 10023, USA; rkhalaja@nyit.edu

**Keywords:** PEDOT, sensor, real-time, nitrate, vapor phase polymerization, conductivity

## Abstract

Nitrate (NO_3_) pollution in groundwater, caused by various factors both natural and synthetic, contributes to the decline of human health and well-being. Current techniques used for nitrate detection include spectroscopic, electrochemical, chromatography, and capillary electrophoresis. It is highly desired to develop a simple cost-effective alternative to these complex methods for nitrate detection. Therefore, a real-time poly (3,4-ethylenedioxythiophene) (PEDOT)-based sensor for nitrate ion detection via electrical property change is introduced in this study. Vapor phase polymerization (VPP) is used to create a polymer thin film. Variations in specific parameters during the process are tested and compared to develop new insights into PEDOT sensitivity towards nitrate ions. Through this study, the optimal fabrication parameters that produce a sensor with the highest sensitivity toward nitrate ions are determined. With the optimized parameters, the electrical resistance response of the sensor to 1000 ppm nitrate solution is 41.79%. Furthermore, the sensors can detect nitrate ranging from 1 ppm to 1000 ppm. The proposed sensor demonstrates excellent potential to detect the overabundance of nitrate ions in aqueous solutions in real time.

## 1. Introduction

On a global scale, monitoring nitrate (NO_3_) levels in groundwater is essential, particularly in developing countries. Common causes of increased nitrate levels worldwide include agricultural practices, parent geological materials, soil-drainage rates, land-use patterns, and aquifer types [1]. In the United States of America, the maximum contaminant level for nitrate in drinking water accessible to the public is 10 mg/L (10 ppm) as nitrate-nitrogen [2]. The maximum contaminant level declared by the World Health Organization (WHO) is nearly equivalent in concentration, which is 50 mg/L as nitrate or 11.3 mg/L nitrate-nitrogen [2]. Evidently, with a clear standard for nitrate levels in water, there is an indisputable need for methods to detect and monitor these levels. High levels of ingested nitrate in the human body can lead to various complications. One significant complication arises when nitrate is converted into nitrite by bacterial enzymes in the digestive system. When this occurs, nitrite can react with hemoglobin (oxyHb) and form methemoglobin (metHb) and nitrate. This causes impairment of oxygen delivery to tissue [3]. If the levels of metHb in the human body increase by 10% above the normal range, which is typically less than 1% of the total hemoglobin concentration, it can lead to clinical conditions such as cyanosis and asphyxia [3].This fatal condition is known as methemoglobinemia and is more likely to occur in infants than in children and adults, who are less susceptible [4]. With the health and well-being of millions of people and entire ecosystems at stake, it is highly desired to develop affordable methods that can monitor nitrate levels in groundwater at high temporal and spatial resolutions.

Current methods for nitrate detection include spectrometers, electrochemical sensors, chromatography, and capillary electrophoresis [5,6,7,8,9,10]. There are numerous advantages to using these devices, including high accuracy, resolution, and excellent repeatability. However, these devices also present significant disadvantages, such as long sample preparation time, latency between data capture, cross-sensitivity, cost, and in some cases, the requirement of high power to conduct these experiments [11,12,13]. Polymer-based sensors have garnered great interest due to their tunability for specific applications, including biosensors, temperature sensors, pH sensors, and ion sensors [14,15,16]. Polymers are low-cost, easy to fabricate and can be deposited on various substrates for sensing system integration [17].

Conductive polymers (CPs) have received extensive attention for their potential in numerous applications that encompass several disciplines of engineering [18,19,20,21]. CPs are polymeric materials composed of individual polymer chains with a conjugated backbone that can be doped with counterions. Conjugated polymers utilize both electron-donating (n-type) and electron-accepting (p-type) dopants, which act as reducing agents and oxidants, respectively, to enhance their conductive properties [22]. Various studies have explored the application of CPs in ion sensing. For instance, Vazquez et al. successfully employed poly(3-octylthiophene) (POT) as potentiometric ion sensors for detecting silver ions [23]. Bomar et al. developed ion-selective electrodes (ISE) based on poly(N-methylpyrrole) to detect nitrate ions in different aqueous solutions [24]. Wang et al. reported the utilization of polyaniline (PANI) in an electrochemical sensor for detecting cadmium ions [25].

Particularly, there has been significant interest in poly(3,4-ethylenedioxythiophene), commonly referred to as PEDOT, due to its remarkable stability, hydrophobic characteristics, and thermoelectric attributes [26,27,28,29]. PEDOT is the outcome of polymerizing 3,4-ethylenedioxythiophene (EDOT) monomers and is a polythiophene derivative [29]. The length of PEDOT chains is relatively short, typically spanning just a few tens of monomer units [30]. Various studies have employed reducing agents and oxidants to orient charges into conjugated polymers such as PEDOT, thus enhancing their conductive properties [22]. While the majority of research has traditionally emphasized the conductivity aspect of PEDOT, there has been a noticeable shift in focus towards exploring its use in sensing applications. For instance, Popov et al. conducted a comparative study on polyaniline (PANI), poly(3,4-ethylenedioxythiophene) (PEDOT) and PANI-PEDOT films in pH-sensing capabilities [29]. Sun et al. created a poly(3,4-ethylene dioxythiophene):poly(styrenesulfonate) (PEDOT:PSS) and chemically cross-linked poly(acrylamide-co-methacrylic acid) hydrogel for strain-sensing applications [31]. Alshawi et al. utilized a sensor based on a platinum electrode (Pt) modified by poly(3,4-ethylenedioxythiophene) and N_α_,N_α_-bis-(carboxymethyl)-L-lysine hydrate (NTA lysine) for determining trace levels of mercury (Hg^2+^), lead (Pb^2+^), and zinc (Zn^2+^) ions in water [32].

In recent studies conducted by Rudd et al. from Evans’ group, a novel application of PEDOT doped with tosylate was reported for sensing nitrate ions in various water samples, including agricultural water from the soil [33]. The sensing mechanism relied on the re-doping process of the electrochemically reduced PEDOT:Tos film with nitrate ions present in the electrolyte. Rudd and colleagues demonstrated that the resulting electrical conductivity of the PEDOT:Tos-nitrate thin film exhibited a direct proportionality to the concentration of nitrate within the electrolyte. Additionally, their PEDOT:Tos films showed favorable selectivity towards nitrate ions when tested in aqueous solutions containing multiple ions. This selectivity arises from the different manners in which anions insert into the PEDOT structure, leading to distinct responses in terms of electrical properties. For instance, nitrate ions insert within PEDOT:Tos to form π-anion-π stacking, whereas for other ions such as chloride anions, the PEDOT polymer chains are π-π stacked with anions situated around the π-π stacked PEDOT sheets [34].

Furthermore, Rudd et al. showed that different copolymers and processing temperatures result in different materials’ electrical conductivities, charge transport behaviors, surface roughness, and chain ordering levels. Through analyzing samples with various properties, they suggested that the sensitivity of the nitrate uptake is dependent on the chain ordering in [x00] plane, surface morphology, electrical conductivity, and charge transport [35]. Later, Shahina et al. demonstrated that PEDOT could detect nitrate via a change in optical properties because of the oxidation of PEDOT [5]. The proposed platform exhibits the change in the optical behavior of the PEDOT layer at the tip of the fiber as it experiences chemical oxidation and reduction [5].

The novel research conducted by Evans’ group has shown the promising potential of the PEDOT:Tos polymer film for low-cost, real-time nitrate sensing in wireless sensor platforms. However, there are several unresolved issues that need attention. Firstly, although the group reported real-time monitoring of nitrate using optical methods, they did not present the real-time electrical conductivity response to ion uptake. To simplify measurement and enhance practicality, it would be preferable to focus on electrical methods. Further studies are needed to demonstrate the film’s real-time electrical properties in response to ion uptake. Secondly, the group identified reproducibility issues in PEDOT, where processing parameters significantly impact the material’s properties and sensitivity to nitrate uptake [5]. While factors such as high electrical conductivity, metallic-like charge transport, increased chain ordering, and smoother morphology contribute to high sensitivity, their interdependence and influence on sensitivity remain unclear. Thorough studies are required to understand these interactions and enhance reproducibility.

In this study, we address the research gaps for PEDOT sensors and investigate the real-time resistivity response of the partially reduced PEDOT:Tos-based resistive sensors to nitrate uptake. We also delve into the investigation of how various polymerization parameters, such as temperature, pressure, and time, influence the sensitivity of the sensor to nitrate. We identify the optimal fabrication conditions that maximize nitrate sensitivity and discuss the influence of these processing parameters on the material’s properties and its subsequent sensitivity to nitrate uptake. With optimal fabrication parameters identified, this nitrate ion-sensitive film has the potential to be integrated into wireless and passive sensor platforms, such as surface acoustic wave (SAW) sensors for passive and wireless sensing applications. This integration opens up opportunities for real-time, non-intrusive detection and monitoring of nitrate ions, enabling efficient and convenient sensing solutions in various applications such as environmental monitoring, agriculture, and water quality management.

## 2. Materials and Methods

### 2.1. Materials and Equipment

3,4-Ethylenedioxythiophene 97% (EDOT), Iron (III) p-toluenesulfonate hexahydrate, n-Butanol, and PEG-PPG-PEG, Mn = 5800 were purchased from Sigma-Aldrich (Sigma-Aldrich Co. LLC., St. Louis, MO, USA). Plain glass microscope slides (75 mm × 25 mm), IPA (isopropyl alcohol), acetone, ethyl alcohol, and DI (deionized) water were purchased from Fisher Chemical (Thermo Fisher Scientific Inc., Waltham, MA, USA). All materials were used without further purification. Required equipment used in this experiment included a spin coater (Brewer Science Inc., Rolla, MO, USA), a hotplate (Brewer Science Inc., Rolla, MO, USA), a VWR 1490 vacuum oven (127 L) (VWR International LLC., Radnor, PA, USA), an AJA ATC-Orion 8 UHV sputtering system (AJA International Inc., North Scituate, MA, USA), an Alpha-Step D-600 profiler (KLA Corp., Milpitas, CA, USA), a VersaProbe II XPS system (Physical Electronics Inc., Chanhassen, MN, USA), a scanning electron microscope (Hitachi 4800 SEM, Hitachi Ltd., Tokyo, Japan), an automated multipurpose X-ray diffractometer (XRD) (Rigaku Corp., Tokyo, Japan), and a Keithley 2401 source meter (Tektronix Inc., Beaverton, OR, USA).

### 2.2. Vapor Phase Polymerization

The PEDOT:Tos thin films were synthesized using the vapor phase polymerization (VPP) method in a VWR 1490 vacuum oven. The glass substrates were cleaned using acetone, IPA, and DI water, and then dried with a nitrogen spray. After that, the electrodes were fabricated by sputtering a 450 nm gold film onto the glass substrates through a shadow mask, using the AJA sputtering system. Following the fabrication of gold electrodes, a 21.3 wt% oxidant solution was prepared by dissolving Iron (III) p-toluenesulfonate hexahydrate in n-Butanol. Subsequently, the working solution was synthesized by combining the 10 wt% PEG-PPG-PEG tri-block co-polymer with the oxidant solution. The working solution was then spin-coated onto the substrate at 1500 RPM for 25 s. The samples were then transferred to the hotplate and baked at 70 °C for 60 s to remove the solvent in the films.

**Figure 1:**
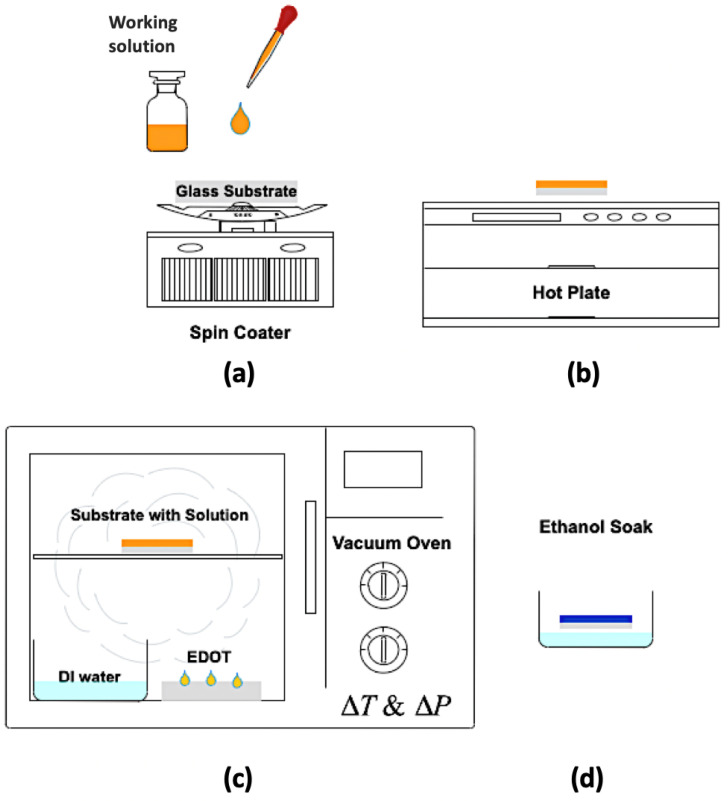
Procedure for the VPP process. First, (**a**) the oxidant solution is spin-coated onto a substrate at 1500 rpm for 25 s and then (**b**) placed onto a hotplate at 70 °C for 60 s to evaporate the solvents. Then, (**c**) the oxidant films are exposed to monomer vapor at a given temperature (T) and pressure (P) inside the vacuum oven. The newly created PEDOT:Tos film (**d**) is then rinsed with ethanol to remove any remaining oxidant and unreacted monomer.

A vacuum oven was then prepared for the sample. DI water and EDOT monomer were placed in the oven. For all variations of this experiment, 0.3 mL of DI water was used. This amount of water was chosen to keep the pressure inside the chamber constant throughout the experiment. To start the polymerization process, the oven was set to 40 °C and pumped down to −97.517 kPa for 1 h to allow the EDOT monomer to saturate the oven. After one hour, the oven was brought back to atmospheric pressure and the samples were loaded. For the vapor phase polymerization process, variations in temperature, pressure, and polymerization time were explored to determine the optimal parameters to enhance the sensor’s response to nitrate. The oven was then set to the desired temperature and pumped down to the desired pressure (less than 4 min of pumping was needed to reach these pressures). After the polymerization, the sensors were soaked in ethanol to remove any unreacted chemicals. The samples were then dried in open air. This process is illustrated in Figure 1.

To investigate the effect of the chamber temperature on the resulting thin film’s physical properties and the response to nitrate ions, the vacuum oven temperature was varied while keeping the pressure and polymerization time constant. This resulted in a ten-degree temperature range at two-degree increments, as indicated in Table 1. Similarly, to study the effect of the chamber pressure, the vacuum oven pressure was varied while keeping the temperature and polymerization time constant. This resulted in a pressure range of 3.386 kPa at 0.677 kPa increments, as indicated in Table 2. Furthermore, the polymerization time was varied while keeping the pressure and temperature constant. This resulted in a 25-min time range at 5-min increments, as indicated in Table 3.

### 2.3. Characterization of the PEDOT:Tos Film

**Figure 2:**
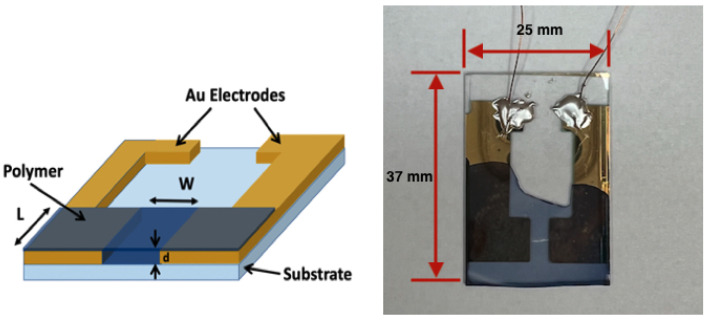
Fabricated sensor configuration which includes gold electrodes attached to a glass substrate with PEDOT:Tos polymer attached and copper wiring attached to the gold electrode using silver paste.

Once the sensors were thoroughly dried, they were characterized for their conductivity. The thickness (*d*) of the polymer thin film was determined using a stylus profiler (Alpha-Step D-600, KLA Corp., Milpitas, CA, USA). To obtain the measurements, a small scratch was made at the center of the film. The stylus profiler then measured multiple points along this scratch, allowing for accurate determination of the film’s thickness by averaging the data. The sensors were wired at the uncovered end of the sensor using a low-cure temperature silver paste and 30 gauge copper wiring. The wired sensor can be seen in (Figure 2). The resistance (*R*) between the electrodes was measured under 2 mV DC voltage supply with a source meter (Keithley 2401, Tektronix Inc., Beaverton, OR, USA). The following equation was used to calculate the conductivity of the thin film:

(1) $$Conductivity=\frac{W}{L\times R\times d}$$

where *L* and *W* are the length and width of the polymer film, respectively.

A scanning electron microscope (Hitachi 4800 SEM, Hitachi Ltd., Tokyo, Japan) was used to analyze the surface topography of the PEDOT material. For this experiment, PEDOT:Tos films were fabricated using the fabrication parameters listed for a polymerization time of 25 min (condition 13) and 50 min (condition 18) listed in Table 3. Using the same samples, an automated multipurpose X-ray diffractometer (XRD) (Rigaku Corp., Tokyo, Japan ) was used to analyze and compare the crystal structure of the samples mentioned. A 2θ range of 4–20° with a 0.2° step at a speed of 5°/min was used to collect the X-ray diffraction patterns.

A VersaProbe II XPS (Physical Electronics Inc., Chanhassen, MN, USA) system was utilized to analyze the surface chemistry of the PEDOT material. This was done to validate nitrate absorption into the thin film at various concentrations. For this experiment, PEDOT:Tos films were fabricated on glass slides using the fabrications parameters listed for condition 18 in Table 3. All samples were submerged in DI water for 24 h before nitrate exposure. Nitrate solutions were prepared with concentrations of 1 ppm, 10 ppm, 100 ppm, and 1000 ppm and separated into individual Petri dishes. Each sample was submerged in the corresponding solution for 24 h. The chemical composition data were obtained by averaging 2 survey scan spectra. Spectra were charge-corrected relative to the aliphatic carbon peak at 285 eV. The atomic percentage of nitrogen (N) was extracted from the XPS survey scan to determine nitrate absorption into each sample. The XPS parameters follow the procedure reported in previous literature [35].

### 2.4. Experiments for Water Absorption and Nitrate Sensing

Both water absorption and nitrate test experiments were performed under ambient conditions, and the resistance between the electrodes was measured with a source meter (Keithley 2401, Tektronix Inc., Beaverton, OR, USA) with 2 mV of DC voltage applied. There were two forms of testing to analyze the effect of water absorption on the thin film’s electrical properties. For the first test, sensors were exposed to the surrounding environment for four months. The resistance was recorded approximately once a week. For the second test, sensors were connected to the source meter and completely submerged in DI water. The source meter recorded the electrical resistance every second until the thin film was completely saturated.

For nitrate sensitivity test, nitrate solutions with concentrations of 1 ppm, 10 ppm, 100 ppm, and 1000 ppm were prepared and placed in separate Petri dishes. The sensor was initially submerged in DI water until the resistance measurement stabilized. Then, the sensor was immersed in the 1 ppm, 10 ppm, 100 ppm, and 1000 ppm solutions, respectively, until stabilization occurred. This process was then repeated in reverse order, starting from 1000 ppm and going down to 100 ppm, 10 ppm, and 1 ppm, respectively.

To assess repeatability, the sensor was tested multiple times using a 100 ppm nitrate solution. Initially, the sensor was fully immersed in DI water to stabilize the readings. Then, the sensor was transferred to the 100 ppm solution until stabilization was achieved. Afterward, the sensor was returned to the DI water. This entire process was repeated for a total of six cycles.

## 3. Results and Discussion

### 3.1. Significance of Water in the Synthesis of PEDOT:Tos

For this study, 0.3 mL of water was used in the VPP process. It is understood and accepted that water plays a crucial role in the VPP process of the synthesis of PEDOT. Water acts as a proton scavenger and is necessary for PEDOT polymerization. The VPP process is not able to produce conductive PEDOT:Tos thin films without the introduction of externally supplied water vapor. This has been proven in the literature [36].

Mueller’s experiments concluded that at least 0.75 mL of water was needed for confluent PEDOT:Tos films to be produced [36]. This is controversial with the amount used in this study. However, the water in the Petri dish is not the only water source available in the experiment. Mueller identified three additional water sources in the VPP process other than water added in a Petri dish. The first source, and the smallest portion of extra water, is located as a hydration shell in the co-polymer (PEG-PPG-PEG). PEG is also known for its hydroscopic properties. Furthermore, the co-polymer prevents the iron salt crystallization in the presence of water and moderates the polymerization rate [37]. The more significant portion of supplemental water comes in the form of free water within the solvent carrier, such as the dissolved water in butanol. Furthermore, water that is coordinated with the metal Fe(III) centers of the oxidant is retained in a usable/accessible form upon introduction of the co-polymer PEG-PPG-PEG [38]. Additionally, the difference in experimental setups can contribute to variations in water usage. In Mueller’s study, water was frozen on a Peltier device within the chamber and later boiled off at the start of polymerization. In contrast, the current study evaporated water in a Petri dish within the chamber prior to and during the polymerization process. The water quantity (0.3 mL) selected for this experiment was carefully determined to fulfill the necessary conditions for polymerization to occur and to sustain the desired pressure within the chamber. To enhance the optimization process in future experiments, the manipulation of water concentration within the chamber holds promising potential as a valuable variable to explore.

### 3.2. Properties of Pristine PEDOT:Tos Film

**Figure 3:**
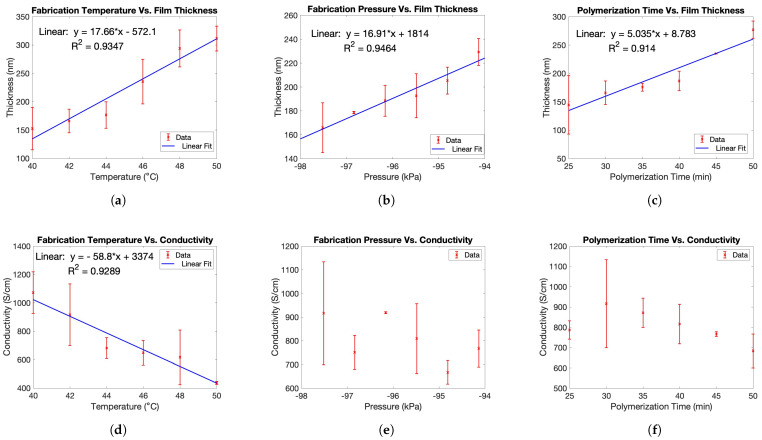
Fabrication temperature vs. (**a**) film thickness and (**d**) conductivity of PEDOT:Tos film. Fabrication pressure vs. (**b**) film thickness and (**e**) conductivity of PEDOT:Tos film. Polymerization time vs. (**c**) film thickness and (**f**) conductivity of PEDOT:Tos film.

In this research, our hypothesis is that three crucial processing factors, temperature, pressure, and time, can influence the morphology, structure, and deposition rate of the PEDOT:Tos films. Consequently, these factors have the potential to alter the material’s response to nitrate ions. Figure 3 shows the effects of the three processing parameters on both the thickness and electrical conductivity of the fabricated film. It is evident based on (Figure 3a) that as the polymerization temperature increases, so does the film thickness. This is noticeable by the increase in thickness from 152.83 nm (40 °C) to 311.50 nm (50 °C). Furthermore, as temperature increased, conductivity decreased. This change is apparent in the decrease in conductivity from 1072.82 S/cm (40 °C) to 436.38 S/cm (50 °C) (Figure 3d). These results validate the findings from previous work [39]. Increasing the VPP temperature results in thicker films because higher temperatures favor the evaporation of EDOT, thus increasing the concentration of the EDOT vapors within the chamber. Therefore, there is a higher concentration of EDOT vapor reacting with the surface of the oxidant solution.

**Figure 4:**
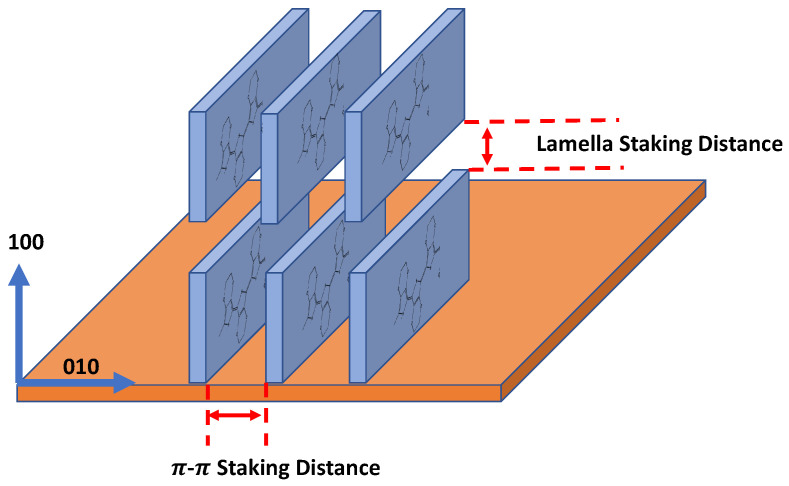
Lamellar stacking of PEDOT chains in an edge-on orientation achieved during the vapor phase polymerization process.

The fabrication temperature in the VPP process has a significant influence on the morphology and the lattice structure of the PEDOT layers. Increased temperatures result in an increase in polymerization rate and structural disorder amongst the chains [40]. This is evident based on the 635.62 S/cm difference between 40 °C and 50 °C (Figure 3d). The crystal structure of PEDOT plays a significant role in charge transport and is greatly influenced by fabrication temperature. The crystalline PEDOT structure is illustrated in Figure 4 and features a lamellar structure involving π-π stacking in [010] direction and regular lamellar inter-chain stacking in [100] direction [41,42]. Compared to the other directional planes, only the lamellar inter-chain stacking order in [100] direction significantly impacts the film conductivity [41]. Wu reported the highest conductivity at 46 °C; however, the results seen in Figure 3d indicate that the optimal temperature is 40 °C to reach higher conductivity. The difference in results is likely due to different concentrations of the working solution and fabrication pressure within the chamber.

Generally, as the pressure decreased, the thickness of the film decreased (Figure 3b). This is evident by the decrease in thickness from 229.9 nm (−94.131 kPa) to 165.95 nm (−97.517 kPa). Increased thickness at higher pressure values results from an increased polymerization rate. It is hypothesized that an increase in pressure can potentially result in higher rates of vaporization for the EDOT monomer, leading to an increased concentration of the monomer in the oven. When there is a higher concentration of EDOT vapor, more interactions occur at the vapor-solution interface, thus resulting in increased polymerization rates and film thickness. As pressure decreased, the polymerization rate decreased, thus decreasing film thickness. Unlike thickness, in this study, there was no clear correlation between fabrication pressure and resulting conductivity (Figure 3e). However, more experiments are needed to validate this conclusion.

**Figure 5:**
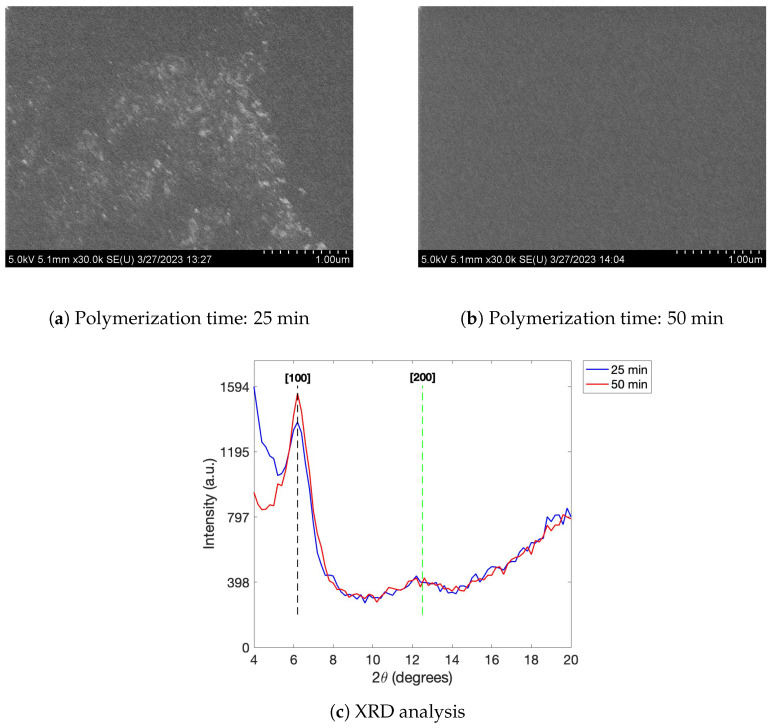
SEM observations for (**a**) 25 min polymerization time and (**b**) 50 min polymerization time fabrication. (**c**) XRD analysis illustrating the molecular order of the PEDOT:Tos chains for both 25 min polymerization time (blue) and 50 min polymerization time fabrication (red).

It is evident that as polymerization time increases, film thickness increases. This trend is represented by the increase from 144.67 nm (25 min) to 277.17 nm (50 min) (Figure 3c). The thickness of the film deposited for 50 min is approximately twice that of the film deposited for 25 min, suggesting that the deposition rate remains consistent. As the deposition time increases, the electrical conductivity initially increases from 786 S/cm (25 min) to 916 S/cm (30 min) and then gradually decreases to 683 S/cm (50 min) (Figure 3f). The increase in conductivity from 25 min to 30 min can be due to the fact that the film has yet to fully develop at 25 min, as a majority of film development occurs during the 30–45-min range, as noted by other works in the literature [43]. The underdeveloped nature of the film is clearly observed in the SEM images depicted in Figure 5a (25 min), where increased roughness, patchiness, and the presence of pinholes are evident. In contrast, the SEM image shown in Figure 5b (50 min) indicates a smoother and more developed film, highlighting the significant improvement in film quality with increased development time. Despite the achieved improvement in film quality (from 25 min to 50 min polymerization time), it is important to acknowledge that this specific parameter may not be the optimal condition for promoting the optimal morphology and conductivity of PEDOT films. This is evident from the observed decrease in conductivity after a 30 min polymerization time. The general decrease from 30 min onwards likely occurs because longer polymerization times lead to higher structural disorder and randomly orientated disconnected islands in the polymer, as noted by Nguyen [40]. These factors limit charge transport and increase the film’s resistance, thus resulting in lower conductivity levels [40,44,45].

X-ray diffraction (XRD) patterns of the 25 and 50 min (Figure 5c) fabricated PEDOT:Tos are illustrated in Figure 5c. The low angle peak (6.2°) represents the edge-to-edge spacing of the PEDOT chains and closely aligns with that of Evans’ group [33,34,35]. Evidently, the peak intensity for 50 min is greater than that of the 25 min fabrication time. One possible explanation for the observed increase in intensity is the thicker film obtained with the sensor fabricated using the 50 min fabrication parameters. Previous studies have suggested that an increase in film thickness can lead to higher peak intensity levels [46]. Alternatively, the increased intensity peak could indicate a more organized arrangement of PEDOT molecules and improved structural characteristics along the [100] direction [35]. However, based on the results presented in this study, the hypothesis suggesting a more ordered arrangement of PEDOT molecules and improved structural characteristics along the [100] direction cannot be confirmed based on the current results. As shown in Figure 3f, there is a decrease in conductivity when comparing the 25 and 50 min polymerization times. Therefore, in this study, the observed increase in intensity peak is hypothesized to be primarily attributed to the increased film thickness rather than improved structural characteristics.

### 3.3. Water Exposure Assessment

**Figure 6:**
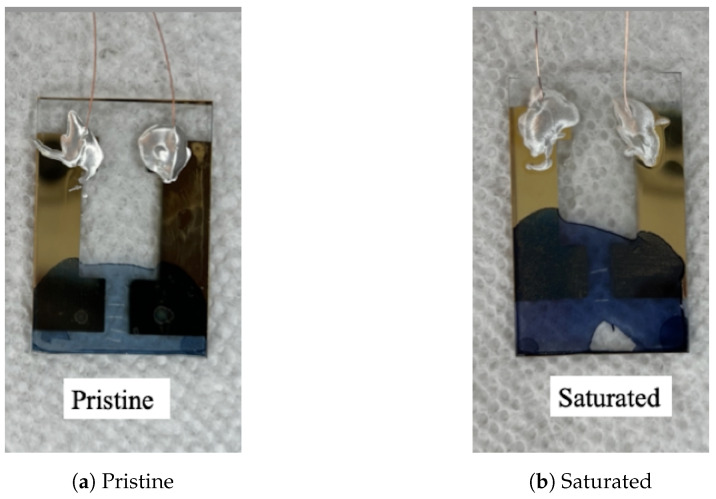
(**a**) Pristine PEDOT:Tos film (light blue) versus (**b**) fully saturated PEDOT:Tos film (dark blue).

The samples were exposed to two different environments to study the effect of water molecules on the film’s electrical properties. As previously stated, there were two forms of testing: exposing the sensors to the surrounding environment for four months and submerging the film in DI water. Both experiments were conducted at room temperature (23 °C). The fabrication parameters for the sensors used in both conditions were: a fabrication temperature of 40 °C, a pressure of −97.517 kPa, and a polymerization time of 30 min. An interesting observation was the sensor’s physical appearance after it was fully saturated. Initially, the film appeared light blue in the pristine condition (Figure 6a) and transitioned to a darker blue after it was completely saturated (Figure 6b).

**Figure 7:**
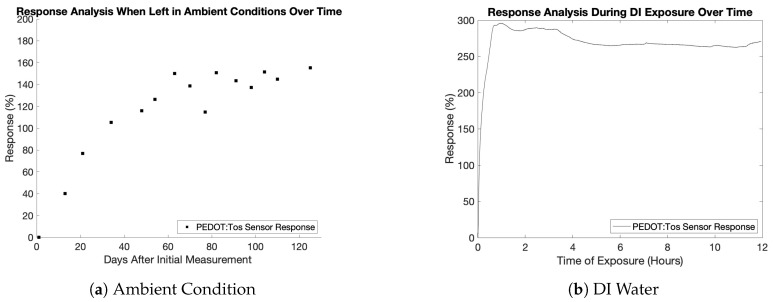
Response of PEDOT:Tos thin-film when (**a**) left in ambient conditions over a four-month period and (**b**) when submerged in DI water.

In both experiments, the sensor was deemed fully saturated once the resistance no longer changed. Figure 7a,b illustrate the sensors’ response to ambient humidity and DI water, respectively. The sensors’ response is defined as the ratio of resistance change after humidity or DI water exposure ($R_{DI/Ambient}$) to the initial pristine resistance ($R_{Pristine}$) and is determined by the following equation:

(2) $$Response=|\frac{R_{DI/Ambient}-R_{Pristine}}{R_{Pristine}}|\times 100\%$$

Resistance changes were measured periodically over four months for the first experiment, where the sensors were exposed to the surrounding lab environment with a relative humidity of between 30 and 50 percent. The sensors showed a gradual increase in resistance over the initial 60 days. Starting from approximately day 60, the sensors reached a saturation point and exhibited a significant increase in resistance, approximately 150% higher than the initial resistance levels. In the second experiment, where the sensors were submerged in DI water, the resistance change was immediately evident and fully saturated within an hour. Likewise, there was about a 250% increase in the film’s resistance.

The significant increase in resistance observed in the PEDOT:Tos thin film can be attributed to two main factors. Firstly, in both ambient humidity and DI water conditions, the resistance increase can be caused by the hydration process, which leads to changes in the material’s morphology and crystalline structure. As mentioned earlier, PEDOT:Tos consists of crystallites (several π-π stacked PEDOT chains) embedded in an unstructured matrix of PEDOT chains [47]. Previous studies have indicated that in the dry state, the pristine PEDOT chains and crystallites are closer together compared to when they are hydrated, forming a mixture of water molecules and PEDOT chains [47]. When the polymer absorbs water and swells, the water molecules occupy spaces between the PEDOT crystallites, increasing the distance between the chains [48]. This increased distance hinders electron hopping, resulting in higher resistance and reduced conductivity. Secondly, when the sensors are submerged in DI water, the increase in resistance can also be attributed to the reduction process in which the tosylate anions diffuse out of the PEDOT film chains. However, this diffusion process does not occur in ambient humidity conditions. Therefore, a significantly higher response was observed when the sensor was submerged in DI water than in ambient humidity conditions.

The results of the above stability assessments in ambient humidity and aqueous conditions reveal that the sensor reaches saturation within one hour and exhibits excellent stability in aqueous conditions over 12 h, establishing its suitability for sensing in such environments. However, the sensor requires approximately two months to achieve saturation in ambient conditions. Given that the reduction process occurs when the sensor is submerged in water, we hypothesize that this process can replace the electrochemical reduction used in Rudd’s study for nitrate sensing [33], offering a simpler alternative approach.

### 3.4. Nitrate Sensing

#### 3.4.1. General Nitrate Sensing and Repeatability

**Figure 8:**
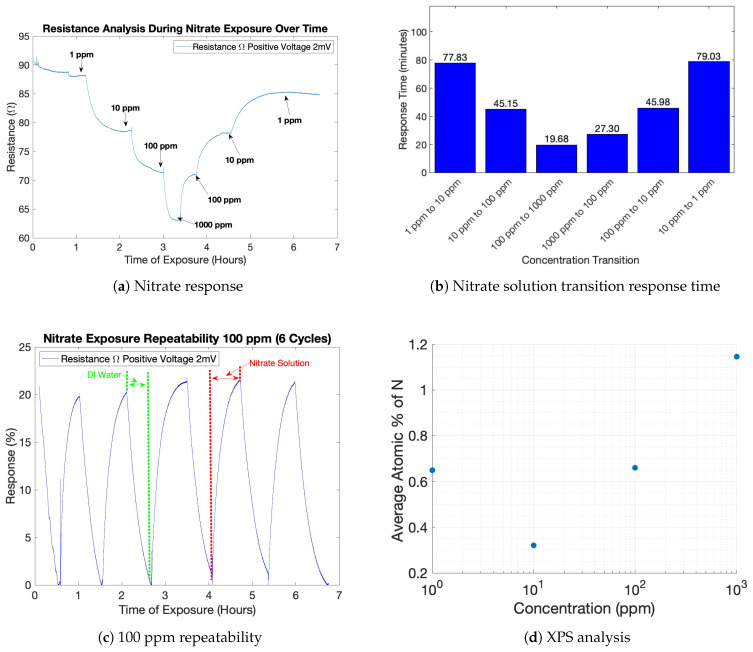
(**a**) Typical response of the PEDOT:Tos sensor’s resistance when exposed to various concentrations of nitrate solutions. (**b**) Response times for each solution transition. (**c**) Typical response of the PEDOT:Tos sensor’s resistance during repeatability test with 100 ppm nitrate solution. (**d**) XPS analysis for various concentrations of nitrate solution.

After the pristine PEDOT:Tos samples were fully saturated from DI water and partially reduced, they were prepared for nitrate testing. The general resistance response to various nitrate concentrations is illustrated in (Figure 8a) and the corresponding response times in (Figure 8b). All sensors fabricated in this study exhibited similar reactions when exposed to various nitrate solutions. Initially, the sensor’s resistance exhibited a decrease as it was exposed to nitrate solutions of increasing concentrations ranging from 1 ppm to 1000 ppm. Subsequently, as the concentrations decreased from 1000 ppm to 1 ppm, the resistance showed an increase. It is worth noting that, despite the reverse order of concentrations, the resistance did not return to its initial value, indicating hysteresis in the sensor’s response. Furthermore, the observed increase in response times when transitioning from higher to lower concentrations was not significantly pronounced, suggesting a relatively minor impact on the overall sensing performance (Figure 8b). Generally speaking, the change in resistance is due to the movement of nitrate anions in and out of the PEDOT:Tos film via diffusion [35]. It is hypothesized that the structure/morphology of the PEDOT:Tos variants play a role in nitrate sensitivity. The preceding sections provide an in-depth analysis of which VPP parameters lead to the fabrication of a highly sensitive nitrate sensor.

Additionally, a repeatability test for 100 ppm was conducted to test the reliability of the sensor’s response to nitrate anions. The results from the six-cycle repeatability test for 100 ppm are illustrated (in Figure 8c). The fabrication parameters for the sensor used in the repeatability test were: a fabrication temperature of 40 °C, a pressure of −97.517 kPa, and a polymerization time of 30 min. The sensor was alternatively exposed to DI water and 100 ppm nitrate solution for six cycles. The sensors’ response is defined as the ratio of resistance change after ion addition ($R_{Nitrate}$) to the initial resistance when the device is exposed to DI water ($R_{DI}$) as determined by the following equation:

(3) $$Response=|\frac{R_{Nitrate}-R_{DI}}{R_{DI}}|\times 100\%$$

Based on the results from this test, the variation in resistance with the device exposed to a 100 ppm solution for each cycle is 20.74 ± 0.38%. Despite not being the optimized sensor configuration, the obtained results exhibit real-time measurement capabilities and demonstrate good repeatability.

#### 3.4.2. XPS Data Analysis

The data collected from the XPS analysis are illustrated in (Figure 8d). The results obtained in this study demonstrate a noteworthy trend. Beyond the 1 ppm concentration, a direct positive correlation emerges between the atomic percentage of nitrogen (N) and the concentration of nitrate (NO_3_). The presence of a 1 ppm outlier in the data could potentially be attributed to subtle discrepancies in the PEDOT:Tos films utilized for each submersion involving different nitrate concentrations. These variations may arise from factors such as slight differences in film thickness or variances in the formation of PEDOT:Tos chains during the fabrication process. Further experiments and more samples are needed to formulate a valid conclusion. Despite the inconsistency in the data, nitrate (NO_3_) uptake is evident.

#### 3.4.3. Fabrication Variations on Nitrate Sensitivity

As previously discussed, the VPP parameters were varied in this experiment to determine the optimal fabrication methodology to produce highly sensitive PEDOT:Tos nitrate sensors. For this experiment, the polymerization time was kept at 30 min and the pressure at −97.517 kPa while the temperature was varied. The goal of this experiment was to determine which fabrication temperature resulted in the highest sensitivity towards nitrate ions.

**Figure 9:**
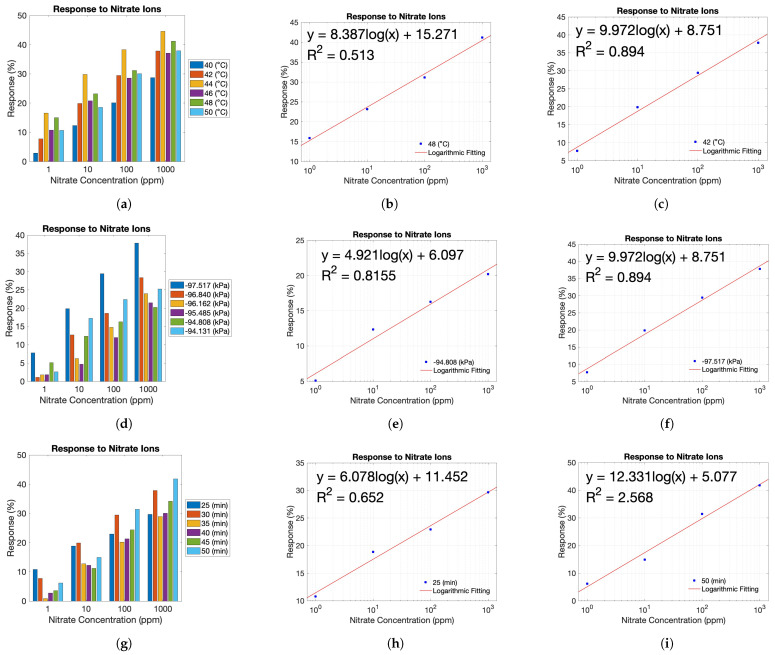
(**a**) Response to nitrate ions while varying fabrication temperature. Additional parameters include a polymerization time of 30 min and a fabrication pressure of −97.517 (kPa). Relationship between the response and the concentration of nitrate solution (**b**) (48 °C), (**c**) (42 °C). (**d**) Response to nitrate ions while varying fabrication pressure. Additional parameters include a polymerization time of 30 min and a fabrication temperature of 42 °C. Relationship between the response and the concentration of nitrate solution (**e**) (−94.808 (kPa)), (**f**) (−97.517 (kPa)). (**g**) Response to nitrate ions while varying polymerization time. Additional parameters include a fabrication pressure of −97.517 (kPa) and a fabrication temperature of 42 °C. Relationship between the response and the concentration of nitrate solution (**h**) (25 min), (**i**) (50 min).

The sensors’ response to nitrate ions can be seen in (Figure 9a). As illustrated in the figure, there were six different batches of sensors fabricated at different temperatures. Based on the results from this experiment, it is apparent that 44 °C had the highest response to nitrate ions in all nitrate solutions 1 ppm to 1000 ppm. The highest response was 44.58% to 1000 ppm. Conversely, the results indicate that 40 °C had the lowest response to nitrate ions in all nitrate solutions 1 ppm to 1000 ppm. The highest response was 28.67% to 1000 ppm. However, although it appears 44 °C is the optimal fabrication temperature, sensitivity toward nitrate ions is more crucial; therefore, the responses were taken as functions of the concentrations of solutions on a logarithmic scale. Once this was established, a logarithmic trendline was inputted into the graph to find the slope of this relationship. This was done for all fabrication temperatures. The greatest sensitivity is determined by the highest values in the resulting slope from this trendline. The results indicated that 48 °C (Figure 9b) was the least sensitive, and 42 °C (Figure 9c) was the most sensitive towards nitrate ions established on the resulting slope. It was determined that 42 °C was the optimal temperature for the proceeding parameter variations.

With the optimal fabrication temperature set at 42 °C, the experiment focused on varying the fabrication pressure while keeping the polymerization time constant at 30 min. The sensors’ response to nitrate ions can be seen in (Figure 9d). Based on the results from this experiment, it is apparent that −97.517 kPa had the highest response to nitrate ions in all nitrate solutions from 1 ppm to 1000 ppm. The highest response was 37.8% to 1000 ppm. Conversely, the results indicate that −95.485 kPa generally had the lowest response to nitrate ions in all nitrate solutions 1 ppm to 1000 ppm. The highest response was 21.45% to 1000 ppm. Again, considering the importance of sensitivity towards nitrate ions, the sensitivity values were calculated as in the previous analysis. The results indicated that −94.808 kPa (Figure 9e) was the least sensitive, and −97.517 kPa (Figure 9f) was the most sensitive towards nitrate ions. It was determined that −97.517 kPa was the optimal pressure for the final variation time.

Once 42 °C and −97.517 kPa were determined as the optimal temperature and pressure parameters, polymerization time was varied. This experiment kept the fabrication temperature and pressure at 42 °C and −97.517 kPa, respectively. The sensors’ response to nitrate ions can be seen in (Figure 9g). Based on the results from this experiment, it is apparent that 50 min had the highest response to nitrate ions in all nitrate solutions 1 ppm to 1000 ppm. The highest response was 41.79% to 1000 ppm. Conversely, the results indicate that 35 min generally had the lowest response to nitrate ions in all nitrate solutions 1 ppm to 1000 ppm. The highest response was 28.93% to 1000 ppm. The sensitivity results indicated that 25 min (Figure 9h) was the least sensitive, and 50 min (Figure 9i) was the most sensitive towards nitrate ions established on the resulting slope. Table 4 illustrates the fabrication conditions alongside their corresponding sensitivity values.

Our optimized sensor demonstrates an impressive sensitivity to nitrate ions, exhibiting approximately a 12% change in resistance response per logarithmic increase in concentration. This sensitivity is significantly higher compared to that achieved by Evans’ group, which showed about a 9% change in sheet resistance per logarithmic increase in concentration. However, our sensor’s responses to nitrate solutions (6% to 1 ppm and 41.79% to 1000 ppm) are comparatively lower than those reported in their study (approximately 38% to 0.2 ppm and 65% to 200 ppm). The variation in response could be attributed to the different reduction methods utilized. It is important to note that concentrations below 1 ppm were not specifically examined in our research. To obtain a more comprehensive understanding of this variation and to explore the performance of our sensors at lower concentrations, further investigations are required. Therefore, within the scope of this study, the sensor demonstrated a Limit of Detection (LOD) for nitrate at 1 ppm, indicating the current lowest reliably detectable concentration. However, it remains to be determined whether the sensor can accurately quantify lower concentrations. As future work, further experiments are needed to establish the Limit of Quantification (LOQ) for nitrate and assess the sensor’s performance in that range.

As mentioned earlier, Bomar et al. presented a sensor utilizing a poly(N-methylpyrrole) based ion selective electrode (ISE) for nitrate ion detection. This poly(N-methylpyrrole) sensor demonstrated a potentiometric response, exhibiting a linear potential change within the concentration range of $5.0\times 10^{-6}$ to 0.1 M nitrate, equivalent to 0.310 to 6201 ppm nitrate [24]. This PEDOT sensor has a narrower range of 1 to 1000 ppm but offers several advantages. Unlike the sensor by Bomar et al., this sensor does not require reference electrodes, simplifying the overall approach. Additionally, the lower sensing range is suitable for applications where a more focused and precise detection of nitrate levels is desired.

Zhang et al. reported an optically-based nitrate sensing platform based on ion-selective membrane (ISM) functionalized chip-scale photonic micro-ring resonators with a sensing range of 1 to 100 ppm nitrate. In order to measure transmission, the device is subjected to light from a tunable external-cavity laser, and the resulting light spectrum is monitored [6]. The PEDOT-based sensor presented in this study provides a more straightforward approach that requires less equipment. Compared to the traditional ion chromatography technique, which typically has a minimum measurable concentration of 0.05 ppm for nitrate, PEDOT-based sensors offer the advantage of real-time monitoring without the need for sample extraction and preparation [33].

Evidently, the optimal fabrication parameters which produce a sensor with the highest sensitivity toward nitrate ions are:

- A fabrication temperature of 42 °C.
- A fabrication pressure of −97.517 kPa.
- A polymerization time of 50 min.

It is hypothesized that the morphology and crystalline structure of PEDOT:Tos influence the resultant sensitivity toward nitrate ions. The described parameters each play a role in both characteristics of the polymer, which is why there is a variation in the sensitivities towards the ion. Previous works have hypothesized that the increased chain ordering in the [100] direction leads to nitrate sensitivity criteria [35,49]. Based on molecular dynamics simulations from other works, it is a possibility that nitrate intercalates within the π-π stacking of VPP PEDOT:Tos at high doping levels [49]. Essentially, the water molecules that previously occupied the space in the π-π stacking are replaced with nitrate via diffusion. Furthermore, based on previous literature published, when there is a higher degree of ordering of the chains, primarily in the edge-on formation, it allows for higher doping of nitrate, which allows a greater opportunity for nitrate intercalation into the π-π stacking of the PEDOT chains [49].

The fabrication temperature of 42 °C is an ideal temperature in that it promotes a slower polymerization rate which results in the higher ordering of the polymer chains. Likewise, the chosen −97.517 kPa has a similar effect on the resulting polymer. The only factor that does not align with this theory is the polymerization time. As discussed earlier, longer polymerization times lead to higher structural disorder and randomly orientated disconnected islands in the polymer. Therefore, it is surprising that this polymerization time (50 min) has resulted in the most heightened sensitivity towards nitrate. It is hypothesized that there is a higher degree of disorder at the top layer of the polymer film than at the bottom and middle layers, which would be produced during the first 30 min. However, it is possible that this disorder allows for more nitrate ions to fit in between the chains, thus increasing the sensitivity towards the nitrate ions. Further validation of this hypothesis is required in order to establish this as a sensible guide toward fabricating a highly sensitive nitrate sensor.

## 4. Conclusions

In conclusion, this study focused on the real-time resistivity response of our partially reduced PEDOT:Tos-based resistive sensor to ion uptake, specifically for nitrate sensing in liquid environments. The findings highlight the sensor’s capability and potential for real-time nitrate detection. By exploring different combinations of vapor phase polymerization processes, we determined the optimal fabrication parameters that yield a sensor with the highest sensitivity towards nitrate ions, exhibiting approximately a 12% change in resistance response per logarithmic increase in concentration. Interestingly, our sensitivity analysis revealed that longer polymerization times led to higher sensitivity, even in the presence of higher structural disorder and randomly oriented disconnected islands within the polymer. This suggests the involvement of additional factors beyond the previously identified factors such as chain ordering level and electrical conductivity, which influence the sensor’s response to nitrate uptake. Further investigation is necessary to unravel these factors.

Future research endeavors may encompass an in-depth exploration of the long-term stability of the polymer, as well as a comprehensive assessment of its lifetime as a sensor. By delving into these aspects, we can gain valuable insights into the polymer’s ability to maintain its structural integrity and functionality over extended periods, thereby enhancing its reliability and durability as a sensing material. In summary, the outcomes of this research contribute to the understanding and advancement of PEDOT as a real-time nitrate sensor. The simplicity and cost-effectiveness of the fabrication method presented here pave the way for its widespread application in diverse industries, including agriculture, food processing, and biotechnology.

## Figures and Tables

**Table 1:** Experimental parameters to produce the different PEDOT:Tos samples using VPP with varying temperature.

Condition	Oxidant	wt%	Co-Polymer	wt%	Pressure (kPa)	Polymerization Time (min)	Temperature (°C)
Condition 1	$Fe{\left(Tos\right)}_{3}$	21.3	PEG-PPG-PEG	10	−97.517	30	40
Condition 2							42
Condition 3							44
Condition 4							46
Condition 5							48
Condition 6							50

**Table 2:** Experimental parameters to produce the different PEDOT:Tos samples using VPP with varying pressure.

Condition	Oxidant	wt%	Co-Polymer	wt%	Pressure (kPa)	Polymerization Time (min)	Temperature (°C)
Condition 7	$Fe{\left(Tos\right)}_{3}$	21.3	PEG-PPG-PEG	10	−97.517	30	42
Condition 8					−96.840		
Condition 9					−96.162		
Condition 10					−95.485		
Condition 11					−94.808		
Condition 12					−94.131		

**Table 3:** Experimental parameters to produce the different PEDOT:Tos samples using VPP with varying polymerization time.

Condition	Oxidant	wt%	Co-Polymer	wt%	Pressure (kPa)	Polymerization Time (min)	Temperature (°C)
Condition 13	$Fe{\left(Tos\right)}_{3}$	21.3	PEG-PPG-PEG	10	−97.517	25	42
Condition 14						30	
Condition 15						35	
Condition 16						40	
Condition 17						45	
Condition 18						50	

**Table 4:** Experimental conditions and the resulting sensitivity to nitrate ions.

Condition	Pressure (kPa)	Polymerization Time (min)	Temperature (°C)	Sensitivity (%/log(ppm))
Condition 1	−97.517	30	40	8.508
Condition 2			42	9.972
Condition 3			44	9.239
Condition 4			46	8.69
Condition 5			48	8.387
Condition 6			50	9.336
Condition 7	−97.517	30	42	9.972
Condition 8	−96.840			8.785
Condition 9	−96.162			7.504
Condition 10	−95.485			9.101
Condition 11	−94.808			4.921
Condition 12	−94.131			7.304
Condition 13	−97.517	25	42	6.078
Condition 14		30		9.972
Condition 15		35		9.171
Condition 16		40		9.113
Condition 17		45		10.502
Condition 18		50		12.331

## Data Availability

Not applicable.
